# Mechanotransduction regulates inflammation responses of epicardial adipocytes in cardiovascular diseases

**DOI:** 10.3389/fendo.2022.1080383

**Published:** 2022-12-16

**Authors:** Xiaoliang Liu, Lei Liu, Junfei Zhao, Hua Wang, Yifei Li

**Affiliations:** ^1^ Key Laboratory of Birth Defects and Related Diseases of Women and Children of Ministry of Education (MOE), Department of Pediatrics, West China Second University Hospital, Sichuan University, Chengdu, Sichuan, China; ^2^ Department of Cardiovascular Surgery, Guangdong Cardiovascular Institute, Guangdong Provincial People’s Hospital, Guangdong Academy of Medical Sciences, Guangzhou, Guangdong, China

**Keywords:** adipocyte, cardiomyocytes, mechanotransduction, cardiovascular diseases, crosstalk

## Abstract

Adipose tissue is a crucial regulator in maintaining cardiovascular homeostasis by secreting various bioactive products to mediate the physiological function of the cardiovascular system. Accumulating evidence shows that adipose tissue disorders contribute to several kinds of cardiovascular disease (CVD). Furthermore, the adipose tissue would present various biological effects depending on its tissue localization and metabolic statuses, deciding the individual cardiometabolic risk. Crosstalk between adipose and myocardial tissue is involved in the pathophysiological process of arrhythmogenic right ventricular cardiomyopathy (ARVC), cardiac fibrosis, heart failure, and myocardial infarction/atherosclerosis. The abnormal distribution of adipose tissue in the heart might yield direct and/or indirect effects on cardiac function. Moreover, mechanical transduction is critical for adipocytes in differentiation, proliferation, functional maturity, and homeostasis maintenance. Therefore, understanding the features of mechanotransduction pathways in the cellular ontogeny of adipose tissue is vital for underlining the development of adipocytes involved in cardiovascular disorders, which would preliminarily contribute positive implications on a novel therapeutic invention for cardiovascular diseases. In this review, we aim to clarify the role of mechanical stress in cardiac adipocyte homeostasis and its interplay with maintaining cardiac function.

## 1 Introduction

Adipose tissue is recognized as a crucial regulator to maintain cardiovascular homeostasis, and the adipose secretes various bioactive products to mediate the physiological function of the cardiovascular system, including adipocytokines, microvesicles, and gaseous messengers, serving as a wide range of endocrine and paracrine effects (1, 2). Recently, accumulating evidence demonstrated that adipose tissue disorders are involved in the pathogenesis of several kinds of cardiovascular disease (CVD), demonstrating a more complex landscape than the previous opinion. Moreover, adipose tissue would present various biological effects depending on tissue localization and metabolic statuses, deciding the individual cardiometabolic risk (3–5). It is known that cardiac adipose tissue is composed of the paracardial fat outside the visceral pericardium and the epicardial adipose tissue (EAT) adjacent to the epicardium (6). In addition to its role in energetic and lipid metabolism, EAT produces amounts of adipokines that freely enter the adjacent myocardium (6). Crosstalk between adipose and myocardial tissue is involved in the pathophysiological process of rapid atrial pacing or atrial fibrillation (AF), which results in regulating adipose tissue accumulation in feedback. It lent itself to that; therefore, abnormal adipose tissue distribution in the heart might yield direct and/or indirect effects on cardiac function.

Additionally, adipose tissue can infiltrate the myocardium and lead to myocardial dysfunction, especially in the right ventricle, which is the dominant phenomenon of arrhythmogenic right ventricular cardiomyopathy (ARVC). Patients with ARVC present the replacement of part of the myocardium by fibrous and fatty tissue with either localized or diffuse myocardial atrophy due to cumulative cardiomyocyte loss. The fibro-fatty scar tissue might progress from the subepicardial muscle layer towards the endocardium, ultimately resulting in transmural lesions with focal or diffuse wall thinning, subsequently leading to cardiac electrical instability (7–9).

Moreover, mechanical transduction is critical for almost all tissues in differentiation, proliferation, functional maturity, and homeostasis maintenance. Generally, a soft extracellular matrix (ECM) or environment is optimal for developing adipose tissue and is seldom applied to high mechanical stress loading. Much evidence demonstrated that soft ECM would ensure adipose differentiation from mesenchymal stem cells (MSCs). Otherwise, the MSCs would present differential features to bone tissue. However, the cardiovascular system bears high pressure with repeated contractile movements, involving amounts of mechanotransduction pathways, namely, focal adhesion kinase (YAP)/transcriptional coactivator with PDZ‐binding motif (TAZ), Mitogen-activated protein kinases (MAPK)/extracellular signal-regulated kinases (ERK), Ras Homolog Family Member A/Rho-Associated Protein Kinase (RhoA/ROCK), and transforming growth factor β1 (TGF-β1)/Smad signaling pathway. And mechanotransduction signaling plays a significant role in the cooperated cells, including cardiomyocytes, endothelial cells, and even adipocytes. Usually, 15–30 kPa stiffness ECM is optimal for cardiomyocytes, while adipocytes require 1–2.5 kPa circumstances. Thus the molecular regulation mechanisms in EAT would be much different from adipose tissue in other locations, which makes mechanotransduction critical to maintaining the homeostasis of EAT.

Therefore, understanding the features of mechanotransduction pathways in the cellular ontogeny of adipose tissue is vital for underlining the development of adipocytes involved in cardiovascular disorders, which would preliminarily contribute positive implications on novel therapeutic interventions for cardiovascular diseases. This review mainly clarifies the role of mechanical stress in cardiac adipocyte homeostasis and its interplays with cardiac function maintenance.

## 2 The cellular origins of cardiac adipocytes

An understanding of the development of cardiac adipocytes (CAs) is essential to demonstrate its involvement in cardiovascular diseases. In adult mouse hearts, adipocytes can be divided into three distinct types according to their anatomical locations. Pericardial cardiac adipocytes (PAT) is situated between the visceral and parietal pericardium, which may be derived from the primitive thoracic mesenchyme and is vascularized by blood from the thoracic vasculature (10). However, the lineage origin and determinative mechanisms of PAT during heart development and disease pathogenesis remains unclear. Most adipocytes are underneath the epicardium, considered as EAT, and most of these subepicardial adipocytes are located in the atrioventricular groove (11). Adipocytes are located in the myocardium, close to the endocardium, and are considered intramyocardial adipocytes. Between approximately postnatal 3 and 4 weeks in mouse hearts, subepicardial and intramyocardial adipocytes could be observed (11). However, these two types of adipocytes originate from different progenitor cells.

Recently, lineage trancing has helped to reveal some fundamental issues on the cellular origins of cardiac adipocytes. Taking advantage of the inducible *Wt1* ^CreERT2^ mouse strain, it demonstrated a large proportion of EAT derives from Wt1-expressing mesothelial cells between embryonic day (E) 14.5 and 16.5 (12, 13). Previous studies showed consistent evidence that subepicardial adipocytes originated from the epicardium (12, 14). Moreover, the mature epicardial cells still reserve the capability to differentiate into adipocytes post-myocardial infarction (13). However, the origins of intramyocardial adipocytes are heterogeneous, and both the endocardium and epicardium contribute to the development of intramyocardial adipocytes during embryonic stages (11). It was identified that the embryonic endocardium first underwent an endothelial-to-mesenchymal transition (EndMT) to form mesenchymal progenitor cells in the endocardial cushion. Then a part of endocardial-derived mesenchymal progenitor cells also migrates into ventricular free walls and septum. Afterward, the mesenchymal progenitor cells could differentiate into intramyocardial adipocytes at the migrated destination (15). Before the appearance of intramyocardial adipocytes at postnatal 3–4 weeks, both endocardial and epicardial derived mesenchymal progenitor cells present the potential ability to differentiate into three essential types of mesenchymal cells (MCs), including fibroblasts, pericytes, and smooth muscle cells (SMCs) during the embryonic stage.

**Figure 1:**
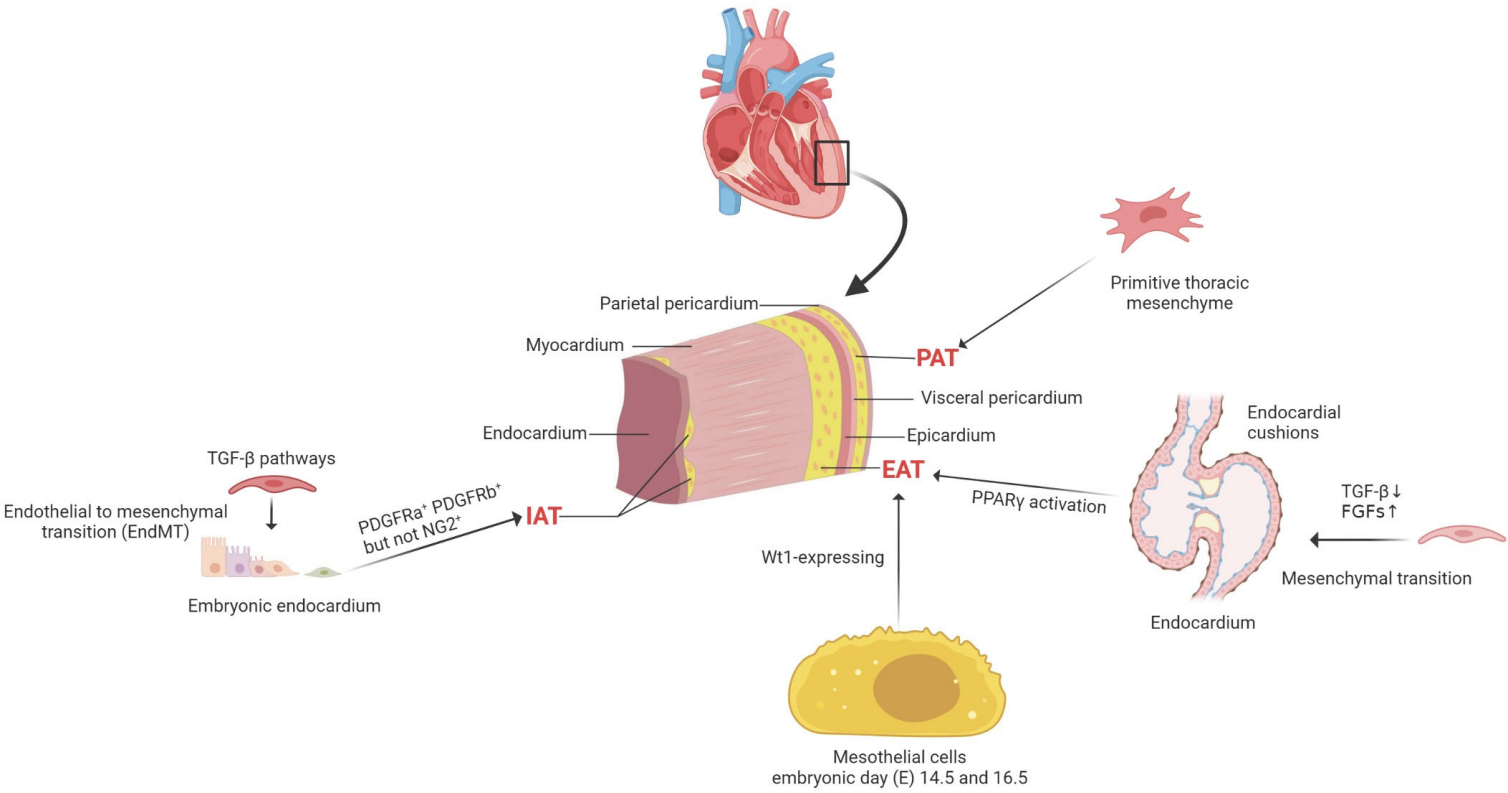
The cellular origins of cardiac adipocytes. PAT is situated between the visceral and parietal pericardium, which derived from the primitive thoracic mesenchyme and is vascularized by blood from the thoracic vasculature. EAT is located between the myocardium and the epicardium, mainly deriving from Wt1-expressing mesothelial cells. IAT is observed in the subendocardium in postnatal mouse hearts; both PDGFRa^+^ and PDGFRb^+^ cells, but not NG2^+^ pericytes, contribute to intramyocardial adipocytes during postnatal heart development and homeostasis. PDGFRa^+^, platelet-derived Growth Factor Receptors a+; PDGFRb^+^, platelet-derived growth Factor Receptors b ^+^; neural-glial antigen 2^+^, NG2^+^; PAT, pericardial cardiac adipocytes; EAT, epicardial cardiac adipocytes; IAT, intramyocardial adipose tissues; TGF-β, transforming growth factor-beta; FGFs, fibroblast growth factors; PPARγ, peroxisome proliferator-activated receptor gamma; Wt1, Wilms tumor gene 1.

Furthermore, the lineage tracing studies showed cardiac MCs contribute to CAs in postnatal development and adult homeostasis. Most intramyocardial adipocytes were derived from the fibroblastic/epicardial marker PDGFRa^+^ (Platelet-derived Growth Factor Receptors a) and the vascular mural cell marker PDGFRb^+^ (Platelet-derived Growth Factor Receptors b) MCs. In contrast, hematopoietic or postnatal endothelial cells did not contribute to cardiac adipocytes. PDGFRa^+^ and PDGFRb^+^ indeed gave rise to adipocytes in adult hearts, while the PDGFRb^+^ MCs contributed to most adipocytes in adult hearts. Moreover, PDGFRa^+^ PDGFRb^+^ periblasts also contribute to adipocytes in adult hearts. Although PDGFRb is expressed in coronary vascular smooth muscle cells (SMCs) (smooth muscle 22 alpha, SM22a^+^ and Myosin heavy chain 11, Myh11^+^) and pericytes (neural-glial antigen 2^+^, NG2^+^), either could develop into cardiac adipocytes. Therefore, it is considered that PDGFRb^+^ NG2^–^ cells are the major source of cardiac intramyocardial adipocytes (16) (**Figure 1**).

## 3 The interplay between the adipose tissue and cardiomyocytes

Adipose tissue is a crucial regulator for myocardial functional maintenance, demonstrating a wide range of endocrine and paracrine effects on the cardiovascular system (1, 2). In the human heart, adipose tissue is mainly located within the atrioventricular and interventricular grooves, surrounding the aortic root and along the coronary arteries’ main branches of the coronary arteries (17). The EAT is close to the myocardium within the right ventricular sidewall and the left ventricular anterior wall (18). EAT shares an unobstructed microcirculation with the myocardium and presents many unique and complex physiological functions, including several metabolic, secretory, thermogenic, and mechanical properties (6, 19, 20). In addition, research has demonstrated that the infiltration and dysfunction of the adipose tissue can, directly and indirectly, impair cardiovascular systems. The cytokines released by adipose tissue by systemic and localized secretion would induce insulin resistance (21), renin-angiotensin-aldosterone system (RAAS) activation (22), lipotoxicity (23), and myocardial interstitial fibrosis (24), which was tightly related to several types of cardiovascular diseases. The adipose tissue-derived cytokines participate in the process of myocardial hypertrophy (25), redox accumulation (26), contractile impairment (27), inflammation invasion, cardiac fibrosis (28), arrhythmogenesis (29), and heart failure (30). Besides, the adipose tissue also directly interacts with the cardiovascular system by releasing amounts of adipocytokines, including adiponectin, leptin, resistin, nitric oxide, interleukins, tumor necrosis factor-a (TNF-a), and other inflammatory factors (1, 31). These bioactive products are crucial for developing CVD with autocrine, paracrine, or endocrine mechanisms (32). It has been proved that the crosstalks between adipose tissue and the heart involve both the coronary artery and myocardium.

**Figure 2:**
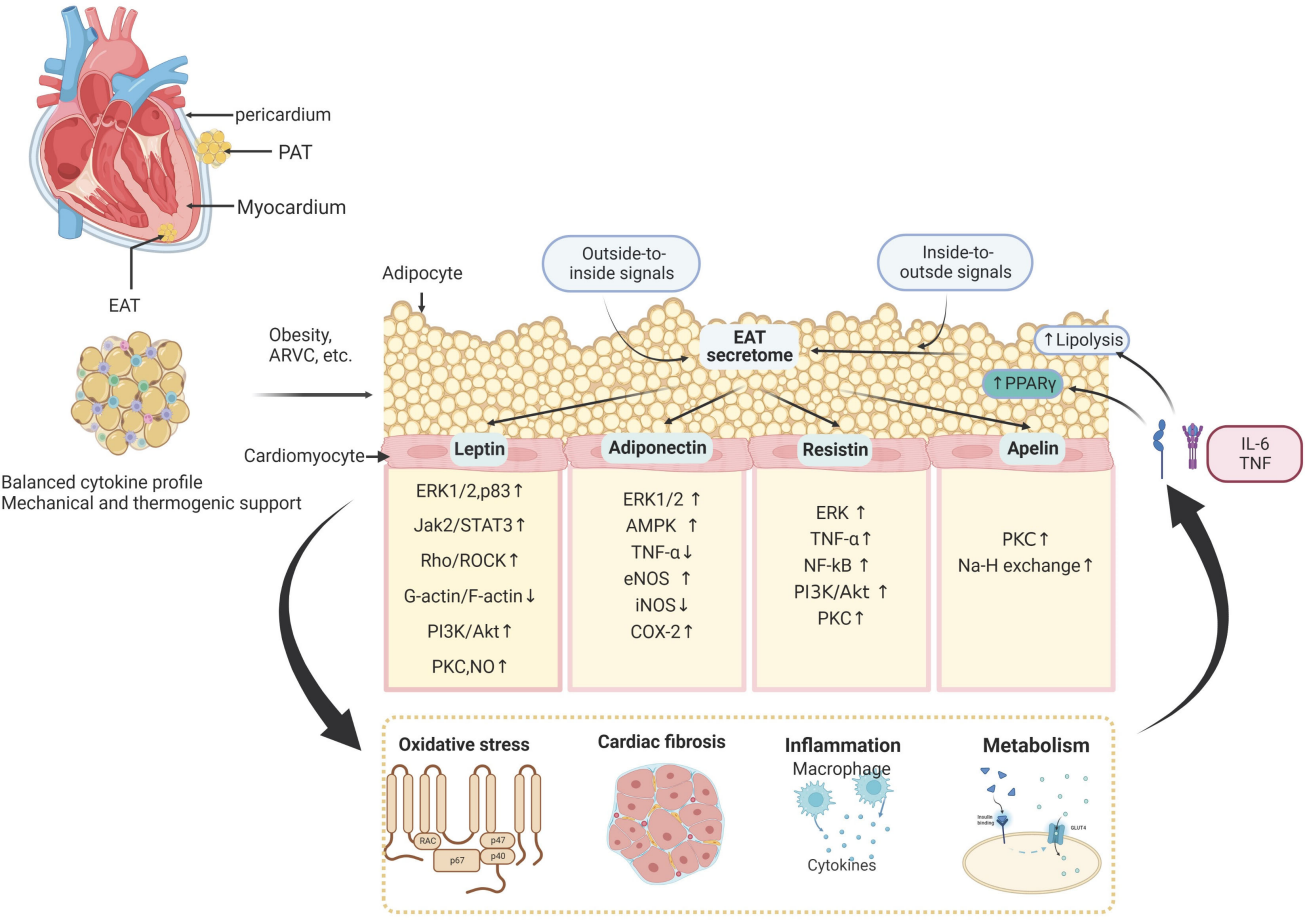
The molecular signaling interplay between the adipose tissue and cardiomyocytes. The epicardial adipose tissue (EAT) lies on the surface of the human heart inside the visceral pericardium, and interacts with adjacent cardiomyocytes and extracellular matrix in a paracrine manner. EAT interacts with the cardiovascular system by releasing amounts of cytokines, including leptin, adiponectin, resistin, apelin, nitric oxide, interleukins, tumor necrosis factor-a (TNF-a), and other inflammatory factors. These EAT-derived molecules are involved in several biological processes in the myocardium, such as oxidative stress, cardiac inflammation, fibrosis, and cardiac metabolism. In addition to these outside-to-inside signals, vascular inflammation and oxidative stress can also affect adipose tissue biology in an inside-to-outside manner. PAT, Pericardial Adipose Tissue; ERK1/2, extracellular signal-regulated kinases 1/2; Jak2/STAT3, Janus kinase 2/signal transducer and activator of transcription 3; Rho/ROCK, Ras homolog gene family member; PI3K/Akt, phosphoinositide 3-kinase/protein kinase B; PKC, protein kinase C; NO, nitric oxide; TNF, tumor necrosis factor; eNOS, endothelial nitric oxide synthase; COX-2, cyclo-oxygenase-2; NF-κB, nuclear factor-κB; AKT, RACα serine/threonine-protein kinase; AMPK, 5′-AMP-activated protein kinase.

Among all the adipose tissue depots, the EAT is most closely associated with myocardial biology since there is no anatomic barrier between the EAT and the myocardium. The mechanisms of paracrine and vasocrine enable communication between the two tissues (1, 33). In addition to its unique anatomical location in the heart, EAT’s metabolic contributions are highly activated by secreting many products with diverse functions. Besides, the metabolic interplays between EAT and myocardium are associated with cardiac metabolic flexibility, energetics, and contractile function (34). Therefore, the homeostasis of EAT biological function is quite sensitive, which would be easily altered (**Figure 2**).

### 3.1 EAT mediates localized and systematic inflammation

Substantial amounts of evidence showed EAT acting as a transducer can adversely mediate the effects of localized and systemic inflammation of the myocardium (35–37). The local and systemic factors of the adipose tissue, such as insulin resistance (IR) (21), renin-angiotensin-aldosterone system (RAAS) activation (22), lipotoxicity (23), and interstitial fibrosis (24), may indirectly contribute to the development of CVD. The adipose tissue can directly affect the cardiovascular system by releasing amounts of adipocytokines, including adiponectin, leptin, resistin, nitric oxide, interleukins, tumor necrosis factor-a (TNF-a), and other inflammatory parameters (1, 31). These bioactive products are crucial to CVD development undergoing an autocrine, paracrine or endocrine mechanism (32). In addition, since the homeostasis and lipogenesis of EAT are tightly associated with systematic body conditions, obesity would change EAT’s biological function and molecular regulation. The epicardial fat thickness might be attributed to systemic inflammation in patients with obese and cardiac fibrosis in patients with chronic heart failure (38, 39). Inflammation can drive adipogenesis, presumably acting as an adaptive mechanism that prevents the deposition of proinflammatory fatty acids in cells other than adipocytes (40). Systemic inflammation also adversely influences the biology of epicardial fat (particularly the perivascular adipose tissue that surrounds the coronary arteries), promoting the expression of a proinflammatory phenotype. Moreover, EAT secretes proinflammatory adipocytokines into the general circulation, which may worsen the systemic inflammatory state. In turn, a positive feedback loop develops as systemic inflammation leads to epicardial adipose tissue accumulation and results in local and systemic inflammation and end-organ dysfunction (35). While the accumulation of EAT is closely associated with impaired myocardial microcirculation, cardiac diastolic filling abnormalities, increased vascular stiffness, and left atrial dilatation in obese people.

### 3.2 EAT influences fatty acid oxidation in cardiomyocytes

Translational mechanistic studies have shown that factors secreted from EAT disrupt fatty acid beta-oxidation in cardiomyocytes, which is their normal major source of energy, accounting for 60–70% of the ATP produced. Chronic systematic inflammation induces more lipid accumulation both in EAT and cardiomyocytes (41), and the overload of FA alters posttranslational modifications of the mitochondrial fission and fusion proteins, including increased ubiquitination of A-kinase anchor protein 121 (AKAP121), dynamin-related protein 1 (DRP1), and proteolytic processing of optic atrophy 1 (OPA1), which are considered as a key part of related molecular mechanisms (42). Analyses of human heart samples showed that mitochondria utilize fatty acid oxidation (FAO) instead of glucose in obese hearts, contributing to metabolic inflexibility (43). EAT Glucagon-like peptide (GLP)-1R was directly associated with fatty oxidation-related genes, while GLP-2R was inversely related to fatty oxidation-related genes (44). Moreover, the concentration of adiponectin decreasing in the EAT could impair mitochondrial OXPHOS capacity (45); and the oxycholesterols closely increases epicardial fat thickness (46).

### 3.3 EAT participates in the regulation of oxidative stress and arrhythmias

Excessive production of mitochondrial reactive oxygen species (ROS) in adipose tissue is known to be an early instigator of adipose tissue inflammation. Increased lipid levels and hyperglycemia can lead to adipocyte mitochondrial dysfunction and ROS production, and this phenomenon has been associated with insulin resistance. At the same time, the abnormal accumulation of localized ROS would transduce mitochondrial dysfunction from EAT to cardiomyocytes. In patients with cardiovascular diseases, there is increased ROS production in EAT compared to SAT and lower catalase levels (47). Subsequently, the increased ROS level may contribute to Ca^2+^ mishandling (48). Intracellular Ca2+ level was elevated, accompanied by the effect of apigenin on adipocyte browning in IL-1ß-treated adipocytes, which might be partly associated with the cAMP/PKA pathway regulated by the activation of transient receptor potential vanilloid 1/4 (TRPV1/4) receptors (49). Moreover, intracellular Ca^2+^ levels increased as TRPV1 was activated by capsaicin, which enhanced the activity of sirtuin 1 (SIRT1) by activating cytosolic AMPK in adipose (50). In addition, EAT infiltrating the atrium has increased the expression of SERCA1 gene encoding for a Ca2+/ATP-dependent intracellular pump involved in oxidative phosphorylation (51). Intracellular and mitochondrial Ca^2+^ is tightly regulated in a narrow range to preserve the overall cellular Ca^2+^ homeostasis and cardiomyocyte contractility. Therefore, it is supposed that Ca^2+^ mishandling might contribute to prolonged diastolic relaxation, decreased fractional shortening, and compromised ejection fraction (52).

### 3.4 EAT regulates myocardial fibrosis

The pro-fibrotic effects of EAT have been identified, which are suggested to be harmful to the cardiovascular system. Research has demonstrated that adipocytokines should be blamed for this pathological process. Activin A, a kind of dominant adipocytokines, would promote atrial fibrosis, which is a member of the transforming growth factor-β (TGF-β) superfamily. Additionally, rat cardiomyocytes demonstrate reductions in contractile dysfunction after prior treated with conditioned media from cultured diabetic human EAT. The decreased insulin-mediated Ser473-phosphorylation AKT signaling and elevated SMAD2 activation (a TGF-β pathway protein implicated in cardiomyocyte fibrosis) have been confirmed, revealing that EAT can affect cardiomyocyte function and remodeling. Moreover, cardiovascular fibrosis could also be developed due to mitochondrial oxidative stress and endoplasmic reticulum (ER) stress activation (53).

## 4 Mechanotransduction in adipocytes

It is increasingly recognized that adipocytes are mechanosensitive and mechanoresponsive cells as mechanical stimuli mediate the adipose differentiation process and function maintains. Dynamic mechanical stimulation loading, likely cyclic stretching or vibration, could suppress adipocyte differentiation progenitor cells, including mesenchymal stem cells (MSCs), preadipocytes, and adipose tissue stromal cells, and several signaling pathways were identified to be involved in the regulation. During the mesenchymal lineage selection, high mechanical stress can be recognized by MSCs and attenuate adipogenic differentiation from MSCs (54) *via* inhibition of GSK3β with subsequent activating β-catenin (55). Also, other research indicates that cyclic stretching would alter the fate of MSCs to adipocytes (56, 57). The inhibition of adipogenesis has relied much on the duration of dynamic loading sessions (55). Besides, Huang et al. (58) found that the outcomes of adipogenesis inhibition depended on the magnitudes of the cyclic mechanical stress on cultured MSCs. In contrast, static stretching might promote adipogenesis (59), and it is believed the different signaling pathways would be involved in adipogenesis in response to cyclic and static stress (60). Furthermore, the examination of static stress loading on adipocytes showed a dual effect on adipogenesis: static stretching accelerated differentiation (60), but static compression inhibited (61). Animal models were also used to evaluate the effects of mechanical stimuli on adipogenesis, presenting dynamic loading suppressing adipogenesis (62–66). This revealed that mechanical stress is critical for adipogenesis, and different types of mechanical stimuli loading demonstrated controversial effects on adipocyte fates (55, 67).

Moreover, in the post-differentiation stage of adipocytes, mechanical stress also participates in maintaining the homeostasis of adipocytes. Expressing lipid droplets and cytoskeletal rearrangements in adipogenesis requires optimal ECM mechanical properties. The increased connective fibers in ECM may generate excessive mechanical forces on adipocytes during several kinds of CVD, especially for cardiac fibrosis. Unlike other adipose tissue, cardiac adipocytes are physiologically exposed to compound mechanical stress, including tensile, compressive, and shear strains/stresses (68).

**Figure 3:**
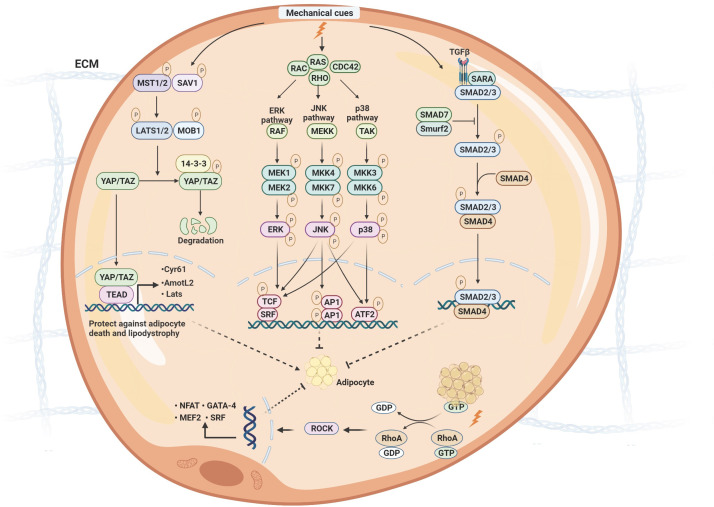
The molecular signaling of mechanotransduction in adipocytes. The molecular signaling of mechanotransduction in adipocytes includes focal adhesion kinase (YAP)/transcriptional coactivator with PDZ‐binding motif (TAZ), Mitogen-activated protein kinases (MAPK)/extracellular signal-regulated kinases (ERK), Ras Homolog Family Member A/Rho-Associated Protein Kinase (RhoA/ROCK), and transforming growth factor β1 (TGF-β1)/Smad signaling pathway. Moreover, mechanical stimuli mediate adipose differentiation. YAP/TAZ signaling pathway enhances adipogenesis; MAPK/ERK, RhoA/ROCK, and TGF-β1/Smad signaling pathway inhibit adipogenesis. ECM, extracellular matrix; Mst, mammalian ste20-like protein kinase; LATS1/2, large tumor suppressor 1 and 2; MOB1, MOB kinase activator 1; SAV1, Sav family containing protein 1; YAP/TAZ, yes-associated protein)/transcriptional co-activator with PDZ binding motif; TEAD, members of the TEA domain; CDC42, Cell division control protein 42; ERK, extracellular signal-regulated kinase;MEKK, MAP kinase kinase kinase; TAK, TGF β-activated kinase; MKK, MAP kinase kinase; JNK, c-Jun N-terminal protein kinases; TCF, ternary complex factor; SRF, serum response factor; AP1, Activator protein 1; ATF2, Activating transcription factor 2; TGF, transforming growth factor β; SARA, Smad anchor for receptor activation; Smurf2, Smad ubiquitin regulatory factor 2; GTP, guanosine triphosphate; GDP, guanosine diphosphate; RhoA, Ras Homolog Family Member A; ROCK, Rho-Associated Protein Kinase.

Furthermore, the mechanical cues on the adipocytes might regulate inflammation and mitochondrial function through various mechanotransduction signaling pathways (69). The fundamental biological role of mechanotransduction signaling is to transduce physical stimuli to molecular pathways and transcriptional regulation. Generally, the differentiation and functional maturation of adipocytes are required mechanical loading, which is also sensitive to mechanotransduction regulation (68). There are two types of mechanical sensors associated with adipocytes, known as biophysical and biochemical sensor mediating pathways. The sensors are located in the membrane to transduce extracellular stress stimulation into intracellular signals. Generally, the role of the physical sensors is to connect the ECM and cytoskeleton, reshape actin proteins, and finally change chromosome structure to influence gene transcription. Biochemical sensors mainly mediate the modification of downstream molecules to transduce signals into transcription regulation, including phosphorylation and ubiquitination (**Figure 3**).

### 4.1 YAP/TAZ signaling pathway

YAP acts as a transcriptional co-factor that is highly associated with TAZ. Both YAP and TAZ interact with the TEA domain (TEAD), which contains family transcriptional factors to induce gene transcription for diverse cellular processes, such as cell proliferation and differentiation. The Hippo pathway negatively regulates YAP and TAZ, regulating organ size and tumorigenesis (70–73). Extracellular matrix rigidity, shear stress, and stretching, regarding mechanical signals, control the activity of YAP and TAZ (74), as well as cytoskeletal tension (74, 75). Summarily, a stiff environment favors YAP nuclear localization and activation, whereas attachment to soft substrates increases cytoplasmic retention of YAP (74).

Stiffness determines the adipogenesis or osteogenesis of MSCs *via* the translocation of YAP. External forces transmit through cytoskeleton reorientation to assist nuclear deformation and molecule transport; meanwhile, signal pathways, including the Hippo, focal adhesion kinase/Ras Homolog Family Member A**/**Rho-Associated Protein Kinase(FAK/RhoA/ROCK), and Wnt/β-catenin, have been evidenced to participate in mechanotransduction. Weakening cell-substrate interactions affect YAP localization and differentiation of adipose-derived stem cells. Allahyari et al. found that these substrates reduce YAP nuclear localization through decreased cell spreading, consistent with reduced cell-substrate interactions and mechanical stress, enhancing adipogenesis. Moreover, the connective fiber content increases in obese adipose tissue, resulting in improved overall rigidity of the adipose tissue, contributing to increased cell death in the obese adipose tissue (76). Additionally, actomyosin-mediated tension has been shown to regulate the thermogenic capacity of brown adipocytes through YAP and TAZ (77).

The activities of YAP and TAZ increased in response to these mechanical changes in hypertrophic adipocytes, and inhibition or knock-down of YAP and TAZ reduced Wnt5a expression by impairing glucose tolerance regardless of diet type. Also, Jin Han et al. demonstrated that YAP is increased in adipose tissue with weight gain and insulin resistance. Disruption of YAP in adipocytes prevents glucose intolerance and adipose tissue fibrosis, suggesting that YAP plays an important role in regulating adipose tissue and glucose homeostasis with metabolic stress. Gao et al. reveal type I collagen inhibit adipogenic differentiation *via* YAP activation *via* increased mechanotransduction stress. And type I collagen inactivates autophagy by up-regulating mTOR activity *via* the YAP pathway. Through the YAP-autophagy axis, type I collagen improves glycolipid metabolism, increases mitochondrial content, enhances glucose uptake, the reduced release of FFAs and decreases intracellular lipid accumulation.

YAP/TAZ were predominantly in the nuclei of human visceral adipocytes from obese subjects, with the up-regulated target genes of YAP/TAZ, likely *Cyr61*, *AmotL2*, and *Lats*. It suggested that the up-expression of YAP and TAZ in adipocytes was accompanied by obesity (78). YAP/TAZ is crucial for adipocyte survival during obesity, and a loss or deficiency of YAP and TAZ could contribute to the development of adipocyte apoptosis and lipodystrophy. The results from Wang et al. indicate that the YAP/TAZ signaling pathway, which TNFα and IL-1β activate under chronic inflammation stimulation, maybe a target to control adipocyte cell death and compensatory adipogenesis during obesity by activation of c-Jun N-terminal kinase (JNK) and AP-1. During obesity, the activated YAP/TAZ in the adipocytes up-regulates anti-apoptotic and downregulates pro-apoptotic factors, such as BIM (Bcl-2 interacting mediator of cell death) (78).

### 4.2 MAPK/ERK signaling pathway

MAPKs are ubiquitous serine/threonine kinases activated by diverse extracellular stimuli, including growth factors, cytokines, and physiological mechanical signals (79–81). MAPKs are essential for transducing signals from the cell surface, regulating diverse cellular behaviors-coordinating development, proliferation, and differentiation. Three MAPK families are well characterized: ERKs, p38 MAPKs, and c-Jun N-terminal protein kinases (JNKs) (82–84). The ERKs family is mainly activated by growth factors, while the p38 MAPKs and JNKs families are primarily activated by cellular stress. The MAPK/ERK pathway is activated by mechanical stress and can control cell behavior in different circumstances and modify cell-ECM interactions (85–89). However, a single stimulus can co-activate the different MAPK pathways, such as crosstalk between p38 MAPK and ERK signaling pathways (90). Several studies have demonstrated that different mechanical loading vibrations, stretch, and extracorporeal shockwaves affect the osteogenic differentiation of adipose‐derived stem cells (ADSCs) (91–94). In human ADSCs, mechanical stretch significantly facilitates proliferation, adhesion, and migration, suppressing cellular apoptosis and increasing pro‐healing cytokines production. For differentiation of human ADSCs, mechanical stretch inhibited adipogenesis and enhanced osteogenesis, regulated *via* the MAPK signaling pathway (95). And Zhang et al. revealed that ERK stimulates the stretch-induced psteogenic differentiation of ADSCs (96). So, the MAPK/ERK signaling was considered an adipogenic pathway. Besides, the adipogenic property of the atrial secretome was enhanced in atrial fibrillation patients by atrial natriuretic peptide-related-activating cGMP-dependent pathway (97).

In maintaining stem cell homeostasis, MAPK participated in the regulation of apoptosis under mechanical stress (98). Moreover, Akutagawa et al. (99) found that the up-regulation of ERK1/2 signaling would inhibit surrounding cellular apoptosis, which provided a novel insight into the mechanisms of the interplay between adipose tissue and cardiomyocytes. Zhao et al. (100) demonstrated that the MAPK/ERK pathway was involved in the thermogenesis and lipolysis by histamine H4 receptor, indicating mechanical stress mediated the metabolic status of adipocytes *via* MAPK signaling.

### 4.3 RhoA signaling pathway

As the cornerstone member of the Ras Homolog Family Member(Rho) GTPase superfamily, Rho A was initially investigated in cancer cells, associating with cell cycle progression and migration (101). RhoA is crucial for maintaining the cytoarchitecture of the cell as a dominant control of actin dynamics. RhoA signaling is also involved in signal transduction and gene transcription regulation to affect physiological functions, including cell division, survival, proliferation, and migration (101, 102). In terms of cardiac remodeling and cardiomyopathies, RhoA is highly dose-dependent. It is essential for maintaining cytoskeletal organization and cardiac homeostasis under mechanical stress (103), such as cyclic stretch and shear stress (104, 105). Su et al. demonstrate that the external forces transmit through cytoskeleton reorientation to assist the nuclear formation and molecule transportation, and the FAK/RhoA/ROCK signaling has been identified as involved in adipogenesis (106). And the proliferation of ADSCs could be activated by β1 and its crucial downstream signaling molecules, namely the small GTPase RhoA and phosphorylated-myosin light chain. Obesity would induce hypoxia in adipose tissue, which creates a diseased phenotype by inhibiting adipocyte maturation and inducing actin stress fiber formation facilitated by myocardin-related transcription factor A (MRTF-A/MKL1) nuclear translocation and the induced RhoA signaling (107). RhoA has been identified to be responsible for cardiomyocyte hypertrophy due to mechanical stress (103). It regulates the development of adipocyte hypertrophy *via* the interaction between adipocytes and cardiomyocytes (60). In contrast, RhoA mediates adipogenesis in ADSCs by underlying substrate stiffness (108). Moreover, stress fiber formation was also observed in adipocytes from HFD-fed mice, prevented by Rho-kinase inhibition. Further, it demonstrated that mechanical stretch could partly associate with the increased activity of Rho-kinase in the mature adipocytes and stress fiber formation, which revealed positive feedback to induce fibrosis (60).

### 4.4 TGF-β1/Smads signaling pathway

TGF-β and related growth factors secrete pleiotropic factors that play critical roles in embryogenesis and adult tissue homeostasis by regulating cell proliferation, differentiation, death, and migration. The TGF-β family members signal *via* heteromeric complexes of type I and type II receptors activating the Smad family of signal transducers (109). Notably, the type I receptors require specific transphosphorylation by the type II receptors before they are activated to bind and phosphorylate their receptor-activated Smad substrates (110). After the activation of the receptor, Smad proteins are phosphorylated by type I receptor kinase at the two carboxy-terminal serine residues and translocate into the nucleus to regulate gene expression, referring to the TGF-β1/Smad signaling pathway (109, 111). TGF-β1 involves the development of different diseases, including cardiac abnormality, cardiac fibrosis, cardiac dysfunction, and cardiac remodeling, as well as cardiac hypertrophy (112, 113).

Regarding cardiac fibroblasts activated by increasing mechanical stress, TGF-β1 has been identified as one of the most relevant pro-fibrotic factors triggering Smad-dependent signaling cascades (112, 114). It is also considered an intrinsic mechanism of mechanotransduction in the regulation of matrix stiffness-induced adipocyte differentiation. Moreover, concerning cardiac pressure overload, fibroblasts act as a protective role through TGF-β1/Smad3 pathway (115), and the activated TGF-β1 would induce adipose fibrosis with elevated extracellular substrates, which generates high static pressure and tissue stiffness to accelerate the cardiac fibrotic process in turn. Wang et al. suggest that an adipokine, orosomucoid, exerts a direct anti-fibrosis effect in adipose tissue *via* downregulated TGF-β1, and orosomucoid is expected to become a novel target for the treatment of adipose tissue fibrosis (116).

The accumulation of TGF-β1 in adipose tissue under overloading mechanical stress induces mesenchymal stem cells to secrete IL-6, and IL-6 polarizes macrophages into the M2 phenotype by presenting an inflammatory environment (117). The inhibition of TGF-β1 in EAT would reduce the inflammation activity in atrial fibrillation-induced abnormal mechanical stress (118). Moreover, obesity and endotoxemia favor the development of adipose tissue fibrosis, a condition associated with insulin resistance, through immune cell Toll-like receptor 4 and TGF-β1 signaling, indicating a tight association between fibrosis and inflammation (119).

## 5 Mechanotransduction regulates adipocytes in cardiovascular disease

### 5.1 Reduced mechanical signaling induced adipogenesis in ARVC

Arrhythmogenic right ventricular cardiomyopathy (ARVC) is a major type of chronic, progressive, heritable myocardial disorder with a broad phenotypic spectrum caused by mutations in genes encoding proteins of cardiac desmosomes (120). It is characterized by a high risk of sudden cardiac death and progressive heart failure, and its phenotypes are enhanced and triggered by strenuous physical activity, while excessive mechanical stretch and load and repetitive adrenergic stimulation are mechanisms influencing disease penetrance (121, 122). In the cardiac tissue of patients with ARVC, fibro-fatty infiltration occurs predominantly in the left ventricle, with the reduction of cellular adhesion, expression of desmosomes, and cell-cell junction proteins in cardiomyocytes. Approximately 50% of patients with ARVC have mutations in genes encoding desmosomes, which, together with integrins and cadherins, contribute to structural mechanoresponsive cytoskeletal elements, such as F-actin, microtubules, and intermediate filaments. Desmosomes maintain the structural integrity of the ventricular myocardium and are also implicated in signal transduction pathways. Mutated desmosomal proteins are thought to cause the detachment of cardiac myocytes by the loss of cellular adhesions and affect signaling pathways, leading to cell death and substitution by fibrofatty adipocytic tissue (121, 123–125). Generally, the mutation or loss of function on cell-cell junction would reduce the mechanical features of cardiomyocytes, which was confirmed by the inactivation of YAP (126). Accordingly, the ECM in cardiomyocytes turns out to be a soft substrate affecting the interstitial cells, adipocytes, and stem cells.

In ARVC, the mechanical stress leads to intracellular signaling changes mainly involving suppression of Wnt/β-catenin and YAP signaling pathways and activation of the EP300-TP53, which drive alternative cell fate, such as fibrotic or adipogenic signaling (127, 128). It is widely recognized that the suppression of Wnt/β-catenin and Hippo pathways involves the altered mechanotransduction and mechanosensing of cardiomyocytes, eventually leading to an arrhythmogenic phenotype (121). β-catenin is translocated into the endoplasmic reticulum from the sites of cell-cell apposition due to low mechanical stress loading. In addition to the abnormal mechanotransduction in ARVC, fibro-fatty infiltrates predominantly in the left ventricle. The adipose tissue infiltration in the heart is often considered a peculiarity of ARVC (120). Partial nuclear translocation of plakoglobin and subsequent suppression of canonical Wnt signaling had been involved in the pathogenesis of fibroadiposis in the right ventricle and its outflow tract, which were considered the predominant sites of involvement in ARVC. Suppression of the canonical Wnt signaling leads to a switch to adipogenesis in the second heart field progenitor cells (129). Also, the reduced mechanical stress would enhance the adipogenesis process by activating PPARγ and C/EBPα, which were negatively regulated by Wnt/β-catenin (130, 131). A series of studies have reported that the Isl1^+^Wt1^+^ myo-adipo progenitors, Isl1^+^Med2c^+^ progenitors, c-Kit^+^Sca1^+^ progenitors, fibro-adipocyte progenitors, MSCs, and epicardial cells could be the origins of adipocytes under lower mechanical stimulation (132). Besides, PPARc modulators rosiglitazone or 13-hydroxy-octadecadienoic acid (13HODE) had been used to shift glycolysis to fatty acid metabolism to model ARVC lipogenesis in human induced pluripotent stem cell-derived cardiomyocytes (hiPSC-CM) (133). Hippo signaling could be activated in ARVC, which phosphorylated YAP protein and inactivated it. And YAP was a co-factor in interaction with β-catenin, participating in adipogenic phenotype. Additionally, a study based on ARVC transgenic mice model demonstrated that the accumulation of cardiac adipose tissue was not tightly associated with the serum level of low-density lipoprotein, indicating the fate alternation of cardiac progenitor or stem cells would be the major cause of adipose tissue accumulation upon low mechanical stress loading (134).

### 5.2 Stiff ECM promoted adipose-associated inflammation in cardiac fibrosis

Cardiac fibrosis refers to myofibroblasts’ collagen deposition in the ECM (128), which can be found in most cardiac pathologic conditions (135, 136). Although in most myocardial diseases, the extent of cardiac fibrosis predicts adverse outcomes. The quiescent cardiac fibroblasts differentiating from ECM-depositing myofibroblasts are considered a milestone of cardiac fibrosis and a vital process of adverse cardiac remodeling. The complex interplay of biochemical signals and mechanical stimuli accounts for this conversion. Releasing neurohumoral mediators, cytokines, and growth factors activated myocardial injury and transduced fibrogenic intracellular signaling cascades (137). The prolonged mechanical cues activate fibroblast persistently with excessive collagens accumulation, leading to a high stiffness ECM. YAP/TAZ has been implicated in fibrotic responses in the heart by accentuating TGF-β-driven activation of Smad 2/3 (138, 139). Moreover, animal models have shown that YAP/TAZ might contribute to activating myofibroblasts and scar formation *via* directly activating effects on fibroblasts and promoting fibrogenic effects in cardiomyocytes (140), or through inhibiting inflammatory activation of a fibrogenic response in EAT (141). Additionally, integrins, adhesion junction mechanoregulation through nuclear β-catenin translocation, and mechanosensitive ion channels are associated with mechanical stimuli in cardiac fibrosis (142).

With enhancing mechanical stimuli, ECM with elevated stiffness was crucial to fibrosis formation and cellular dysfunction in adipocytes, while fibrosis’s feedback amplification through ECM stiffness develops subsequently (143). These increased mechanical stimuli altered intracellular biochemical signaling to affect adipocytes’ cellular behaviors. The stiffness of ECM determined the adipogenesis, cell size of adipocytes, and the status of lipids metabolism (144). Pathologically, ECM mechanics could affect transcriptional changes in gene expression and subsequent production of ECM-related proteins, further driving adipose tissue fibrosis (145). The ECM alteration inhibits the adipocyte’s ability to accumulate and release lipids, and subsequently, adipocyte dysfunction occurs through collagen type I and VI (76, 146).

With regard to the lack of ECM plasticity, adipocytes present with ectopic lipid deposition decreased lipolysis and adipokine secretion, and increased expression/production of cytokines (IL-6, G-CSF) and fibrotic mediators (LOXL2 and the matricellular proteins THSB2 and CTGF) (147), which promotes myocardial disarray, cardiac fibrosis (32). During cardiac fibrosis, stiff ECM induces the production of pro-inflammatory and pro-fibrotic cytokines or adipokines in EAT, which enhances the localized inflammation in cardiac fibrosis (148). Besides, dysfunction of adipocytes has been identified under exceeding pressure overloading. And exosomes isolated from Ang II-stimulated adipocytes promoted cardiac fibroblast activity. Su et al. (149) reported that miR-23a-3p level was significantly increased in exosomes derived from Ang II-challenged adipocytes and serum exosomes from Ang II-infused mice, and adipocyte-derived exosomes mediated pathologic communication between dysfunctional adipose tissue and the heart by transporting miR-23a-3p. In another study, activation of ADRB3 in adipocytes offers cardiac protection through suppressing exosomal iNOS and inflammation activity, and adipocyte-derived exosomes from ADRB3 knock-out mice accelerated Ang II-induced cardiac fibroblast dysfunction (150). The stimulated adipocytes perpetuated inflammation and fibrosis by producing IL-1β and transforming growth factor TGF-β1, indicating a relevant role in fibrotic remodeling (151). The ECM remodeling caused adipocytes’ mitochondrial disorder, and restoring mitochondrial function would inhibit the ability of transplanted ADSC and inflammation cytokines releasing to reduce cardiac fibrosis (152). Also, the reduction of adipose tissue could ameliorate cardiac fibrosis (153).

### 5.3 Mechanical stimuli-stressed adipocytes mediated metabolic disorder in heart failure

Mechanotransduction is crucial to maintain cardiac function, accounting for suitably sensing and responding to mechanical loads which trigger structural, signaling, and functional alterations due to various cellular processes (154). In addition to cellular and molecular signaling pathways involving heart failure (HF) (155, 156), cardiac mechanotransduction attributing to biomechanical stress is also critical (157). Previously, most studies have focused on the molecular mechanism and impacts of mechanotransduction loading on cardiomyocytes. Currently, as the biological function of EAT in CVD has been identified, the mechanotransduction regulation on adipocytes has been demonstrated. First, excessive mechanical stress would lead to cardiac hypertrophy and heart failure. Upon volume overload, the bio-physical strain and shear forces affect cardiomyocytes and chamber walls and subsequently increase cardiomyocyte length, leading to eccentric hypertrophy and chamber dilation (154, 158, 159). Chronic pressure overload exerts biomechanical fiber stress on the heart and its associated cells (158, 159). In terms of the heart undergoing altered wall and/or tissue stress, cardiac mechanotransduction participates in a basic biological mechanism, determining the adjustments and modifications (160, 161).

Second, failing hearts present abnormal biomechanical features. In HF with preserved ejection fraction (HFpEF) patients, incompatible myocardial contraction induced inflammation in the coronary micro-vasculature and heart, which increased cardiomyocyte resting tension and the end-diastolic wall stress (162–164). In HF with reduced ejection fraction patients, myocardium remodeling (155, 156), such as left ventricle dilatation, wall thinning, ventricular shape changes, myocardial hypertrophy, and myocardial fibrosis, contribute to enhanced biomechanical loading and lead to the end-diastolic wall stress due to subsequently exerting biophysical cues on myocardial tissue and EAT (154). In addition, gene expression is activated by biomechanical stress responding to the alteration of cellular and wall stress in the heart (159, 165). The role of myocardial ECM as a mechanical scaffold that preserves cardiac geometry and facilitates force transmission seems intuitive; however, matrix macromolecules and myocardial cells can also interact with each other. In the pathogenesis of HF, prolonged pressure overload triggers fibrosis and perturbs cardiomyocyte relaxation, increasing myocardial stiffness and causing diastolic dysfunction. Persistent pressure overload eventually results in dilative remodeling and systolic dysfunction. Evidence demonstrates that pressure overload contributes to fibrillar collagens deposition in pressure-overloaded hearts. The cardiac ECM is subsequently affected, increasing passive stiffness by altering mechanical properties of the ventricle and critically regulating inflammatory, fibrotic, and hypertrophic cellular responses. In contrast to pressure overload in the heart, volume overload led to interstitial collagen loss and, remarkably ECM degradation. In response to volume overload, the mechanical stretch may increase oxidative stress and activate an autophagic degradation of procollagen in volume-overloaded hearts.

Along with mechanical stress loading, the metabolic status of adipocytes is a significant determinant of macrophage inflammatory output. Macrophage/adipocyte fatty-acid-binding proteins act at the metabolic and inflammatory pathways interface. Both macrophages and adipocytes are the sites for active lipid metabolism and signaling (166). Moreover, during HF process, chronic adrenergic is stimulated, resulting in aberrant adipose tissue lipolysis, which is mediated by adipose triglyceride lipase (167). In animal models of HF, increased circulating FFA and attenuated insulin response were observed (168). Lipid metabolism in HF is characterized by the downregulation of enzymes of β-oxidation of FA and other mitochondrial enzymes, suggesting an imbalance between myocardial FFA delivery and utilization (169–171).

In contrast, mechanical unloading of the failing heart can increase the adipocyte size and reduce the macrophage infiltration in the heart (172). Additionally, Thiele et al. (173) identified a specific set of fatty acids liberated from adipocytes under isoproterenol stimulation (palmitic acid, palmitoleic acid, and oleic acid), which induced pro-apoptotic effects in cardiomyocytes. And Atglistatin significantly blocked this adipocytic fatty acid secretion. Additionally, Shen et al. (174) revealed a positive impact of adipose-PGC1-α on distal organ systems, with beneficial effects on HO-1 levels, reversing obesity-linked cardiometabolic disturbances by uncoupling protein 1 (UCP1), fibroblast growth factor 21 (FGF21), and pAMPK signaling, with a reduction in inflammatory adipokines. Emerging evidence demonstrated that EAT might guide the therapeutic decision in diabetic patients as drugs such as metformin, glucagon-like peptide-1 (GLP-1) receptor agonists, and sodium-glucose cotransporter 2 inhibitors (SGLT2i) have been associated with attenuation of EAT enlargement (175). Mitochondrial dysfunction would also participate in pressure overloading myocardial injuries, and Hou et al. (176) suggested that UCP1 knockout aggravated ISO-induced myocardial damages by inhibiting AMPK/mTOR/PPARα pathways. Moreover, the p38 MAPK signaling participated in ectopic lipid deposition and metabolic dysfunction with impaired transcripts for oxidative phosphorylation, tricarboxylic acid cycle, and fatty acid metabolism. The impaired metabolism enhanced cardiac inflammation involving neutrophils, macrophages, B- and T-cells, while knock out of p38 MAPK significantly reduced adipose function and inflammation activities, indicating p38 MAPK mediated heart-adipose-immune cell crosstalk.

### 5.4 The impact of adipose tissue under mechanical changes during myocardial infarction/atherosclerosis on recruiting immune cells

The sudden ischemic death of myocardial tissue is referred to as acute myocardial infarction (AMI). Cardiac ischemia induces profound aberrant metabolic and ionic channel activity in the affected myocardium and consequently leads to rapid depression of systolic function. The biochemical profile and compositions of the ECM dynamically change, which is critical for regulating key cellular events during the three processes of infarct healing, including the inflammatory phase, the proliferative phase, and the maturation phase. First, the inflammatory cascade is triggered by alarmins released by dying cells and contributes to the development of infarct healing (177). Second, the phagocytes infiltrate the affected myocardium and clear dead cells and matrix debris by activating anti-inflammatory pathways to inhibit cytokine and chemokine signaling. Undergoing the activation of the renin-angiotensin-aldosterone system and the release of TGF-β, fibroblasts transform into myofibroblasts and promote ECM protein deposition (178). Infarct healing is intertwined with the geometric remodeling of the chamber, characterized by dilation, hypertrophy of viable segments, and progressive dysfunction (178). After AMI, the loss of cardiomyocytes and the dysregulation of ECM homeostasis results in impaired cardiac function and heart failure (178, 179). Therefore, cardiac mechanotransduction is altered since the myocardial ECM as a mechanical scaffold preserves cardiac geometry and maintains cardiac function. Optical coherence elastography showed the infarcted heart features a fibrotic scar region with reduced elastic wave velocity, decreased natural frequency, and less mechanical anisotropy at the tissue level after MI, suggesting lower and more isotropic stiffness (180). In the MI animal model, studies showed that reduced graphene oxide (rGO)/silk yields the ability to promote cardiomyocyte structure formation and functions because it can mimic the natural cardiac ECM and provide mechanical support for recovery after MI (181). Further, rGO/silk remarkably reduces the expression of the canonical TGF-β/Smads signaling pathway and YAP/TAZ in the infarcted heart (182).

Moreover, atherosclerosis plays a critical role in the pathophysiology of MI since atherosclerotic plaques impair vascular function and can lead to arterial obstruction and tissue ischemia, and the rupture of an atherosclerotic plaque within a coronary artery can result in an acute myocardial infarction (183). The atherosclerotic plaques promote the recruitment and entry of cells of the innate immune system into heart tissue, prompting the release of cytokines and aseptic inflammation (184, 185), which affect endothelial dysregulation in the arterial wall. The mechanochemical events also contribute to atherosclerosis development by transforming the proatherogenic mechanical stimulus of blood flow-low and low/oscillatory arterial wall shear stress in the biochemical reactions in endothelial cells and the interplays with adipocytes (186).

The evolution of atherosclerotic lesions and heart disease has been attributed to paracrine signaling between EAT and adjacent coronary vessels and myocardium (187). Even though the epicardium is responsible for the repose period in the adult heart, MI can contribute to epicardial thickening, fetal gene reactivation, and the epicardial progenitors differentiating into fibroblasts and myofibroblasts (188). Previous studies found that the paracrine signals regulate the behavior and fate of injury-activated epicardium since vascular endothelial growth factor A was delivered to the myocardium during MI. Regarding MI, insulin-like growth factor 1 receptor (IGF1R) signaling is a critical pathway for facilitating EAT formation by promoting epicardium-derived cells differentiating into adipocytes (189). Moreover, IGF1 may also associate with the expansion of other visceral fat depots, which share common developmental origins with EAT. The IGF1-driven progenitor fate switch is operative only within a brief time window after MI since IGF1 activation did not stimulate adipogenic differentiation of progenitor cells in the absence of MI. The requirement for MI in combination with IGF1 to stimulate progenitor differentiation into adipocytes may result from hypoxic conditions and MI activation of progenitors (189). So, MI induced adipose proliferation and accumulation in the heart. Then, the deposited adipose tissue recruited and activated immune cells leading to enhanced inflammation in MI, Horckmans et al. (190) infarcted mice also had larger pericardial clusters, and 3-fold up-regulated numbers of granulocyte-macrophage colony-stimulating factor-producing B cells within pericardial AT, but not spleen or lymph nodes.

Furthermore, lipolysis of EAT increased significantly after MI. Removal of EAT improved cardiac function, in part, by weakening the inflammatory response. Accordingly, Gomez et al. (191) showed that a subpopulation of cardiac inflammatory macrophages emerged from myeloid cells of adipose tissue origin and played a detrimental role in cardiac remodeling and function after MI. Diabetes abolishes the ability of adipose tissue-derived myeloid cells to populate the infarcted heart. The production of the adipokine hormone leptin and adipocyte expansion mediated the conversion of monocytes to macrophages post-MI and was involved in the process of angiogenesis (192). The inhibition of PPARγ of ADSCs caused a significantly larger number of M2-polarized macrophages in the infarct area with a significantly longer duration (193).

## 6 Maintaining cardiac function by targeting adipose tissue

Multiple experimental studies have reported that cardiovascular health might benefit from therapeutic interventions targeting adipose tissue. Nonetheless, a safe and cost-effective intervention targeting adipose tissue remains to be further explored. Simple lifestyle interventions and common medications can improve adipose tissue function with substantial cardiovascular benefits.

Several studies showed that cardiovascular health in patients with diabetes might significantly benefit from antihyperglycaemic medications, such as glucagon-like-peptide 1 (GLP1) agonists (194), dipeptidyl peptidase 4 (DPP4) inhibitors (195), and sodium-glucose cotransporter-2 inhibitors (SGLT2i) (196). Additionally, the adipose tissue produces angiotensinogen and angiotensin II, possibly activating the RAAS (197). Thus, the pharmacological intervention targeting the RAAS in the adipose tissue might partly mediate the beneficial effects of angiotensin-converting enzyme inhibitors, angiotensin II-receptor blockers, mineralocorticoid-receptor antagonists, or novel therapeutic agents (198). In addition, other medications might cooperate with brown adipose tissue activation to facilitate the beneficial effects of the medication. Likely, atorvastatin favors the hepatic uptake of lipoprotein remnants due to the brown adipose tissue activation, which results in the lipid-lowering and anti-atherogenic effects of atorvastatin (199). Methotrexate might restore eNOS phosphorylation and endothelial function by decreasing pro-inflammatory cytokines in PVAT and increasing adiponectin expression (25).

Moreover, cardiac remodeling and dysfunction resulting from obesity can be reversible after weight loss (200, 201). Calorie restriction improves vascular insulin sensitivity and reduces age-related pro-inflammatory cytokine production (202). Isocaloric intermittent fasting prevents obesity-related metabolic dysfunction through increased adipose tissue thermogenesis and fasting-mediated periodic upregulation of VEGF in adipose tissue, which is linked with M2 macrophage polarization and adipose tissue browning (203). The male rat model finds that aerobic exercise can restore the anticontractile vascular effect of PVAT and reduce the expression of the inducible isoform of NOS in PVAT by comparing with no exercise, which might be attributed to decreasing circulating insulin, leptin, and TNF levels (204). Compared with dieting alone, exercise-induced weight loss is more likely to improve circulating adipokine profiles and insulin resistance (205). Encouragingly, the randomized, controlled clinical trials demonstrate the beneficial effects of exercise without calorie restriction present with fat reduction, insulin resistance (206), and increasing adipokine profile (205, 206). Exercises with high-intensity interval training substantially reduce total adipose tissue and VAT mass and increase cardiorespiratory fitness in obese children (207, 208). However, simple activities, such as active commuting, can also lead to meaningful weight loss (209).

## 7 Conclusions

The adipose tissue is a dynamic organ involved in various pleiotropic interactions with the cardiovascular system. As such, adipose tissue is increasingly recognized as a crucial regulator of cardiovascular health and a driver of CVD pathogenesis. Moreover, the adipose tissue has striking biological variability depending on its location and metabolic state. Inappropriate adipose tissue distribution induces systemic lipotoxicity and insulin resistance and results in the development of comorbid conditions, such as cardiovascular disease. Therefore, maintaining mitochondrial homeostasis, normal mechanotransduction, and prompt oxidative stress is a potential therapeutic strategy for cardiovascular diseases associated with adipose tissue.

## Author contributions

XL, LL, JZ, and HW reviewed the literature and contributed to manuscript drafting; XL contributed to the figures in this manuscript. YL, JZ, and HW were responsible for the revision of the manuscript for important intellectual content. All authors contributed to the article and approved the submitted version.
